# Léiomyosarcome du pancréas: une tumeur de mauvais pronostic - cas clinique et revue de la littérature

**Published:** 2012-07-12

**Authors:** Said Ait Laalim, Fatim Zahra Hijri, Imane Kamaoui, Karim Ibn Majdoub Hassani, Imane Toughai, Khalid Mazaz

**Affiliations:** 1 Département de chirurgie générale B. CHU Hassan II, Fès, Maroc; 2 Département d’oncologie médicale. CHU Hassan II, Fès, Maroc; 3 Département de radiologie. CHU Hassan II, Fès, Maroc

**Keywords:** Léiomyosarcome pancréatique, immunohistochimie, chirurgie, pronostic, leiomyosarcoma, immunohistochemistry, surgery, prognosis

## Abstract

Le léiomyosarcome (LMS) primitif du pancréas est une tumeur mésenchymateuse rare (0,1% des tumeurs pancréatiques), seulement 47 cas ont été publiés la littérature depuis 1951. Le diagnostique est basée sur l’immunohistochimie. C’est une tumeur très agressive et souvent découverte à un stade avancé. Nous rapportant le cas d’un patient de 40 ans qui présentait un léiomyosarcome primitif du pancréas de la queue du pancréas avec envahissement de la rate et la partie gauche du colon transverse. Une spléno-pancréatectomie caudale carcinologique avec une colectomie segmentaire était réalisée. Des nodules péritonéaux ont apparus 2 mois après l’intervention au niveau du site opératoire et le paient est décédé 3 mois en postopératoire. Le LMS primitif du pancréas doit être évoqués devant les volumineuses tumeurs du pancréas. Une chirurgie carcinologique est le seul traitement curatif. Le pronostic de ces tumeurs est mauvais et la médiane de survie de ces tumeurs ne dépasse pas 2 ans.

## Introduction

Le léiomyosarcome (LMS) pancréatique est une tumeur mésenchymateuse maligne rare ne représentant que 0,1% des tumeurs pancréatiques [1]. Une revue de la littérature révèle uniquement 47 cas publiés à ce jour. Le diagnostique est rarement posé en préopératoire avec un pronostic qui reste sombre. Nous rapportant le cas d’un patient avec un léiomyosarcome de la queue du pancréas localement avancé décédé 4 mois après une spléno-pancréatectomie caudale et colectomie segmentaire à visée curative.

## Patient et observation

**Figure 1:**
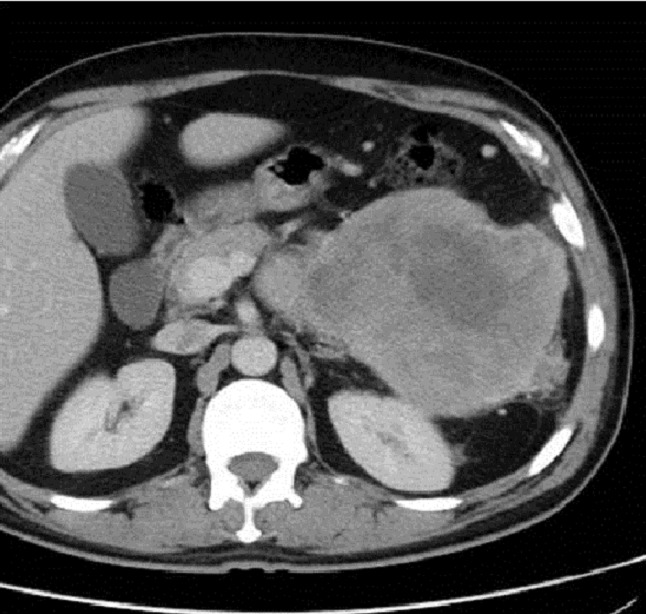
Scanners abdominaux en coupe axiale montrant une volumineuse masse tumorale de l’hypochondre gauche hétérogène mesurant 10 cm de grand axe

Patient de 40 ans sans antécédents pathologiques particuliers, présentait depuis 2 mois des douleurs de l’hypochondre gauche et des lombalgies gauches. L’examen clinique était sans particularité. Une échographie abdominale était demandée et qui montrait la présence d’un processus tissulaire de l’arrière cavité des épiploons de contours polylobés, hétérogène, vascularisée au doppler couleur. On a complétée par un scanner TAP qui montrait une volumineuse masse hétérogène de la queue et du corps du pancréas, envahissant le colon transverse et mesurant environ 20×14×12cm de diamètre avec multiple foyer de nécrose tumorale (Figure 1). Le bilan pré-thérapeutique était sans particularité, les marqueurs tumoraux étaient normaux et une prophylaxie d’une éventuelle splénectomie était réalisée par vaccination anti-pneumocoque et hémophylus influenza. L’exploration chirurgicale trouvait la présence d’une volumineuse tumeur de la queue du pancréas mesurant environ 20 cm de grand axe, de consistance dure, d’aspect multinodulaire avec envahissement de la rate et la partie gauche du colon transverse. Une spléno-pancréatectomie caudale avec une colectomie segmentaire était réalisée en bloc avec anastomose colo-colique termino-terminale. Les suites opératoires étaient marquées par l’installation d’une fistule pancréatique à bas débit, asséchait spontanément.

**Figure 2:**
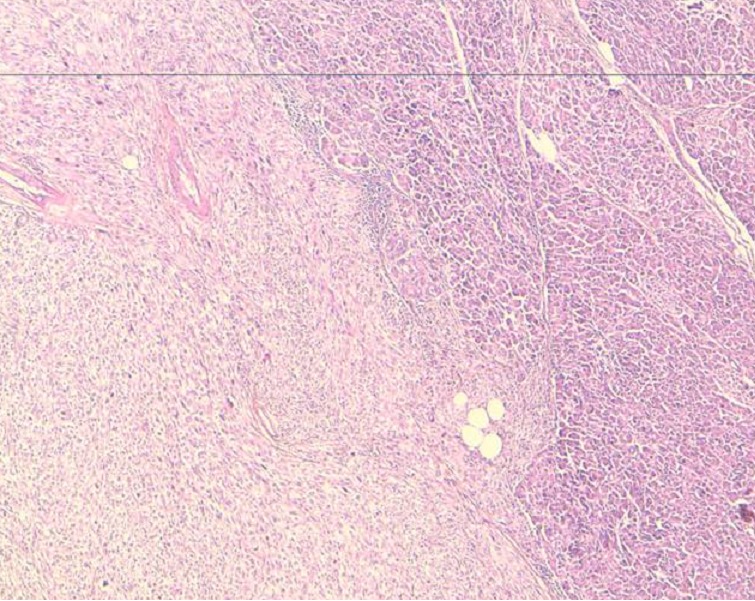
Histologique - parenchyme pancréatique infiltré par une prolifération sarcomateuse fusocellulaire faite de faiseaux entrecroisés avec présence de nombreuses figures de mitoses (HES 5x 2)

**Figure 3:**
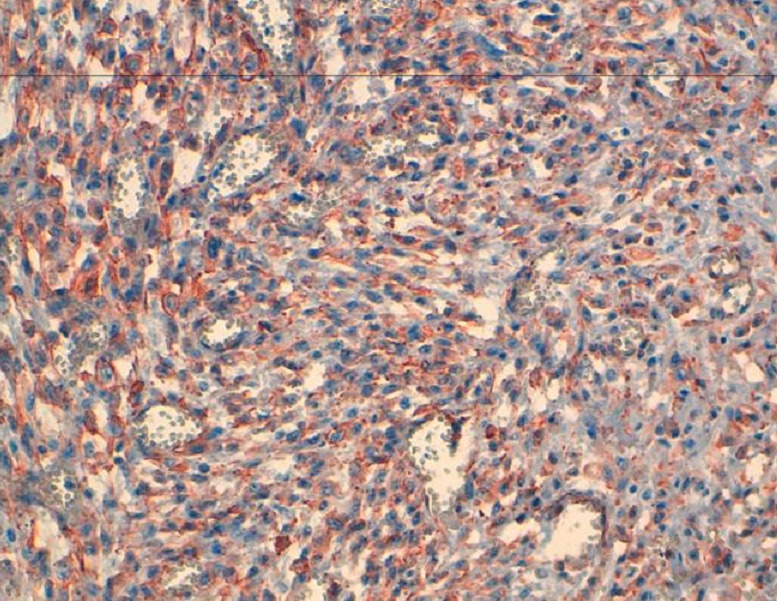
Histologique - étude immunohistochimique (IHC) montrant des cellules tumorales expriment la h-Caldesmone confirmant ainsi la nature léiomyosarcomateuse de la prolifération tumorale. (IHC h caldesmone)

L’examen anatomopathologique de la pièce opératoire montrait la présence d’une prolifération tumorale maligne indifférenciée pancréatique à cellules fusiformes, Il s’y associe un index mitotique important (Figure 2). De larges foyers de nécrose tumorale étaient notés sans emboles vasculaires ni engainement périnerveux. Les Limites de résection pancréatique étaient saines et il n’y avait pas d’envahissement à l’étude histologique du hile splénique, de la rate et du colon. Le curage ganglionnaire trouvait 13 ganglions non envahis. A l’immunohistochimique, les cellules tumorales étaient fortement marquées par les anticorps anti actine musculaire lisse et anti H-caldesmene. Les marqueurs épithéliaux: CK AE1/AE3, CK7 et CK20 étaient négatifs ainsi que la PS 100 et le CD 117 (Figure 3). En conclusion l’aspect histologique et immunohistochimique était en faveur d’un léiomyosarcome primitif du pancréas. Le patient quittait l’hôpital à J + 15.

**Figure 4:**
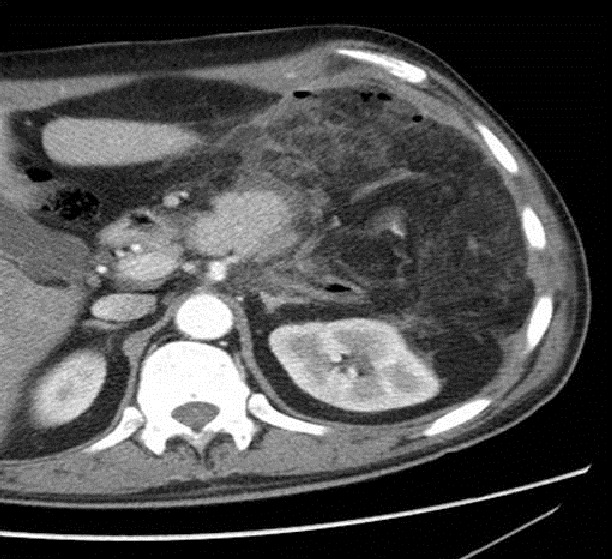
Scanners abdominaux en coupe axiale contrôle post opératoire immédiat montrant un site opératoire vide

Après discussion multidisciplinaire une chimiothérapie adjuvante était proposée pour notre patient à base de Docetaxel-Gemcitabine. Le bilan pré-chimiothérapie incluant une TDM TAP réalisait 15jours en post opératoire était sans particularité (Figure 4).

**Figure 5:**
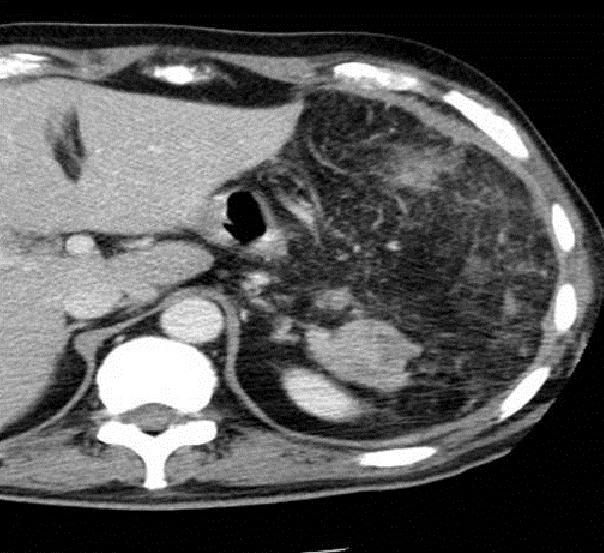
Récidive tumorale sur le site opératoire avec apparition 2 mois en post opératoire d’une masse de 3 cm au niveau du site opératoire

Deux mois en post opératoire le patient était réhospitalisée pour une douleur intense de l’hypochondre gauche résistante aux traitements antalgiques habituels. Une TDM était réalisée et qui montrait la présence d’une masse tumorale de l’hypochondre gauche d’environ 3cm de diamètre avec un aspect de sarcomatose péritonéale localisée au niveau de l’hypochondre gauche (Figure 5) Une exploration chirurgicale était réalisée et qui montrait l’aspect d’un blindage de l’hypochondre gauche. Le patient est décédé après 3 mois de la résection tumorale.

## Discussion

Le léiomyosarcome est une tumeur maligne rare d′origine musculaire lisse qui peut se développé dans n′importe quel organe ou un tissu qui contient du muscle lisse et représente moins de 1% de tous les cancers et 2%–9% des sarcomes [2]. Il est le plus souvent localisé dans l′estomac et l′intestin grêle et peut également être couramment constaté dans le gros intestin, l′utérus, et le rétropéritoine [2]. Léiomyosarcome pancréatique primaire est extrêmement rare et seulement 47 cas ont été rapportés dans la littérature (Tableau 1). Certains auteurs sont sceptiques concernant l’origine pancréatique primitive de la tumeur, car il s′agit d′un site inhabituel pour les tumeurs mésenchymateuses [3, 4]. Les léiomyosarcomes originaires d′autres organes tels que l′estomac, du duodénum, et les organes rétropéritonéaux envahissent souvent le pancréas, simulant une tumeur primitive du pancréas. Le diagnostic de léiomyosarcome pancréatique est confirmé par exclusion des tumeurs de voisinages. Dans notre cas la tumeur siégeait au niveau de la queue du pancréas, l’examen histologique du colon réséqué avec la tumeur montre que ce dernier est indemne de toute prolifération tumorale et que l’origine du LMS est pancréatique.

**Tableau 1:** Léimyosarcome (LMS) primitive du pancreas: literature mondiale (47 cas – présent cas inclus)

Nombre de Cas	Année	1er auteur	Age /Sexe	Symptômes cliniques	Siége/extension tumorale	Conduite thérapeutique et résultats
1	1951	Ross	80 M	Perte de poids, masse épigastrique	Tous le pancréas; métastases multiples	Autopsie
2	1956	Berman	47 M	Ictére	Tête;5.5 cm; Pas de métastases	DPC; En vie: 12mois
3	1957	Feinberg	14 M	Épigastralgie	Tête;11 cm; Pas de métastases	DPC
4	1970	Oyamada	47M	masse épigastrique	15 cm	Non-résécable
5–9	1973	Baylor	Mediane:51 M:3, F:2	–	Localisée:1; Localement avancée:1; Métastases:3	Résécable: 1 Non résécable: 4
10	1976	Carda A. P	56/F	Vomissement	Tous le pancréas	Gastrostomie; DCD: 9 mois
11–12	1981	Heerden	–	–	–	DPC
13	1981	Ishikawa O	44 M	Épigastralgie	Tête; 8 cm, Pas de métastases	DPC; Méta. hépatique. DCD: 4ans.
14	1982	Tulha	28/F	Perte de poids	Tête; 20 cm	DPC; Métastases hépatique et pulmonaire 15 mois après.
15	1990	Murata K	55/F	–	Queue; Pas de métastases	Pancréatectomie caudale
16	1991	Takashima	68 M	–	Tête, 10 cm	DPC; Méta. hépatique 18mois après
17	1991	Lakhoo K	68/M	Perte de poids, masse épigastrique	Corps: 17 cm; Pas de métastases	PD, gastrectomie et colectomie transverse. Vivant 2 ans après.
18	1993	de Alava E	71/M	Épigastralgie Perte de poids	Corps:3.6 cm; Pas de métastases	Pancréatectomie
19	1993	Russ PD	67 M	–	Corps-queue; 10 cm, métastases hépatique	Non résécable
20	1994	Ishii H	66/M	Découverte fortuite	Queue;14 cm; metastases multiples	Non résécable
21	1994	Sato T et al	53/F	masse épigastrique	Corps;25 cm; Pas de métastases	PD
22	1994	Peskova	68/F	Moelena	Tête;15 cm; localisée	DPC; En vie: 3ans
23	1995	Aranha GV	45/F	Épigastralgie	Corps; 3 cm; localisée	PD; DCD 9 mois
24	1995	Hamamoto	55/ F	–	Queue, 5.5 cm	PD; DCD 15 mois
25	1997	Shimizu M	49 F	–	Tête, 15 cm; méta. multiples	Non résécable; DCD:3 mois
26	1997	Owen et al	40/M	Douleur abd.	Tête;6 cm; Pas de métastases	DPC; En vie: 10 ans
27	1998	Chawla S	45/F	Douleur abd.	Tête, 9.2 cm métastases hépatique et pulmonaire	Non résécable; En vie: 19 mois
28	1998	Zalatnai A	57/M	–	Tête, 6 cm métastase hépatique	Non résécable; DCD:7 mois
29	1998	Paciorek M	63/F	–	Corps, 2 cm	PD
30	2000	Ferlan-M. V	57/F	–	Corps, 12 cm	PD; DCD: 5jours
31	2000	Machado M	52/M	–	Tête, 7.5 cm	DPC; En vie: 24 mois.
32 – 35	2000	Srivastava DN	49/ M 38 /M 45 /M 41 /M	– – – –	Corps –Queue, Corps –Queue. Méta. péritonéale Tête, Tête, 3.5 cm	PD Non résécable; DCD: 3 mois Non résécable DPC; DCD: 6 mois.
36	2001	Deveaux1PG	44 F	masse épigastrique	Tête, 7 cm	DPC; En vie: 4 ans
37	2001	Boyer. C	61/F	Douleur abd.	Tête, 5 cm. Méta. hépatique	Non résécable
38	2001	Nesi et al	76 /M	Fièvre	Queue, 8 cm.	PD; DCD: 12 mois
39	2002	Aihara H	25/F	–	Corps, 3.5 cm	Résection locale; En vie: 24 mois
40	2002	Komoda H	52/F	–	Tête, 2cm	DPC; En vie: 48 mois
41	2003	Sharma AK	28/F	Épigastralgie	Tête	Non résécable
42	2008	Shams UlIM	73 /M	masse épigastrique	Corps, 10 cm. Méta. hépatique	Non résécable; DCD: 3 mois
43	2009	Rifki Jai S	41/M	Douleur abd.	Tête, 12,7×11 cm	DPC; DCD: 12 mois
44	2010	Nicole D	83/F	Douleur abd.	Queue, 8 cm	SPD; En vie 8mois.
45	2010	C. Nobili	71/F	Épigastralgie, moelena	Corps–Queue, 6×5cm. Métastases	SPD et multiples métastasectomie; En vie 18 mois
46	2011	Peter Ambe	52/M	Épigastralgie,	Tête	–
47	2011	Young Hoe Hur,	70/F	Pesanteur abdominale	Tête, 4.5×5 cm	DPC; Méta. Hépatique, 6mois après. DCD 22 mois.
Notre cas	2012	S. Ait laalim	40/M	Épigastralgie, vomissement	Corps–Queue, 20 ×14×12	SPD et colectomie transverse; DCD: 3 mois.

DPC: Duodeno-pancréatectomie céphalique; DCD: Décédé; SPD: Spléno-pancréatectomie distale; PD: Pancréatectomie distale; Méta.: Métastases.

D’après les cas publiés dans la littérature (Tableau 1), l’âge des patients varie de 14 à 83 ans avec une moyenne d’âge de 53 ans et une légère prédominance masculine (54,3% Vs 45,7%). La taille de ces tumeurs varie entre 2 et 25 cm avec une moyenne de 9,4cm. La tête du pancréas est le siège de prédilection de ces tumeurs et se voie dans 54,8% des cas (Tableau 2).

**Tableau 2:** Léiomyosarcome primitif du pancréas (LMS). Répartition du siège de la tumeur selon la littérature mondiale

Siège	Fréquence	Pourcentage valide
Tête du pancréas	23	54,8
Corps du pancréas	10	23,8
Queue du pancréas	7	16,7
Tout le pancréas	2	4,8
Totale	42	100
Non décrit	6	

La symptomatologie est aspécifique et souvent tardive ce qui explique la découverte de ces tumeurs à un stade avancé [5]. L’imagerie est aussi peu spécifique [6]. Il s’agit parfois de lésions kystiques de grande taille, parfois de masses solides hétérogènes témoignant de composantes hémorragiques et nécrotiques [7]. L’IRM abdominale peut aider au diagnostic en révélant une masse hétérogène en hyposignal T1 et hypersignal T2 [7]. Ces tumeurs posent le problème de diagnostic des tumeurs hypervasculaires du pancréas qui fait discuter principalement certaines tumeurs endocrines, des tumeurs épithéliales papillaires solides et kystiques, des cystadénomes, cystadéno-carcinomes ou des pseudo-kystes. Le diagnostic devrait pouvoir être évoqué devant le caractère hyper vasculaire et partiellement nécrotique qui est habituel dans ces tumeurs mésenchymateuses quel que soit leur siège [8].

Histologiquement, le LMS du pancréas se développe à partir des cellules musculaires lisses des canaux pancréatiques ou de la paroi des vaisseaux intra-pancréatiques [9]. L’analyse immunohistochimique a une grande valeur dans le diagnostic des LMS du pancréas, en mettant en évidence la positivité des marqueurs des cellules musculaires lisses (MSA, desmine, caldesmone, et la vimentine). La négativité des marqueurs des cellules épithéliales (cytokératine, EMA, CEA) permet d’éliminer l’origine épithélial de ces tumeurs et également la négativité à l’immunomarquage avec les anticorps antiCD 117, CD 34 et proteine S-100 permettaient d’éliminer le diagnostic de tumeur stromale ou schwannome [10, 11]. Dans notre observation l’étude immunohistochimique a montrée que les cellules tumorales étaient fortement marquées par les anticorps anti actine musculaire lisse et anti H-caldesmene confirmant ainsi la nature mésenchymateuse de la lésion. Les marqueurs épithéliaux CK AE1/AE3, CK7 et CK20 étaient négatifs ainsi que la PS100 et le CD 117.

Les léiomyosarcomes d’origine digestive, caractérisés par l’absence de l’antigène CD 117 (ou c-kit), sont, autant que les sarcomes des tissus mous, extrêmement peu sensibles à la chimiothérapie [12]. À ce jour, la radiothérapie joue son rôle dans le traitement préopératoire des sarcomes utérins et des extrémités; en revanche, il n’y a pas d’indication systématique dans les formes rétro-péritonéales [13]. Pour ces raisons, le consensus actuel est que le seul traitement à visée curative est l’exérèse chirurgicale complète (résection R0). Dans notre cas la tumeur siégeait au niveau de la queue du pancréas elle mesurait 20×14×12 cm et semblait envahit le colon transverse et la rate et chez qui une spléno-pancréatectomie caudale associée à une colectomie segmentaire était réalisée.

Les LMS du pancréas sont réputées comme étant des tumeurs de très mauvais pronostics, et ceci est du essentiellement au retard diagnostic et à l’agressivité de la tumeur. Au moment du diagnostic de la lésion pancréatique, cette dernière s’avère non résécable dans 37% des cas (Tableau 1). Le suivi des malades a été rapporté dans seulement 26 cas. La survie médiane chez ces patients était de 16,50 mois (6 – 24 mois). Plusieurs facteurs sont considérés comme prédictifs d’agressivité: la taille, le nombre de mitoses par champs à haut grossissement, le grade de l’atypie cellulaire, la présence de myofibrilles [14]. Pour notre patient on a été impressionné par l’évolution extrêmement rapide de la tumeur après une résection chirurgicale jugée carcinologique, qui a causée la mort du patient en moins d’un mois et dont la survie n’a pas dépassée 3 mois.

## Conclusion

Le LMS primitif du pancréas est une tumeur rare, hautement maligne et de pronostic sombre due surtout au retard diagnostic. En l’absence actuellement de chimiothérapie et de radiothérapie efficaces, la résection tumorale complète R0 peut offrir à ces patients une survie plus prolongée.

## Consentement

Les auteurs déclarent avoir reçu le consentement écrit de la famille du patient pour reporter ce cas.
